# Evaluation of maternal health literacy and its impact on pregnancy outcomes: A longitudinal study in a tertiary care hospital, Karachi

**DOI:** 10.1186/s12884-026-09512-4

**Published:** 2026-07-01

**Authors:** Komal Samir, Aiesha Ishaque, Muhammad Imran, Tushar Subash, Arun Subash, Naveed Gianchand

**Affiliations:** 1 Indus Hospital and Health Network, Karachi, Pakistan; 2 People’s Primary Healthcare Initiative (PPHI), Karachi, Pakistan; 3 Aga Khan University, Karachi, Pakistan; 4 Dow Medical College, Karachi, Pakistan

**Keywords:** Maternal health literacy, Prenatal care, Postnatal complications, Gestational diabetes mellitus, Preterm delivery, Low birth weight

## Abstract

**Introduction:**

Maternal health literacy (MHL) plays a crucial role in impacting pregnancy outcomes, access to healthcare, and the general health of mothers and newborns. In Pakistan, where health challenges such as population growth, maternal education deficits, and poverty persist, health literacy is a growing concern. Despite progress, with 58% general health literacy recorded in 2019, gaps remain, particularly in maternal health awareness.

**Objectives:**

This project intends to assess the level of maternal health literacy among pregnant women and to examine its association with selected maternal and neonatal outcomes at a tertiary care hospital in Karachi.

**Methodology:**

This longitudinal observational study was conducted at The Indus Hospital, Karachi. A total of 386 pregnant women (gestational age > 28 weeks) were enrolled and interviewed using non-probability consecutive sampling during the third trimester, then prospectively followed through labor and the immediate postpartum period to document maternal and neonatal outcomes via clinical records. MHL was assessed using a validated questionnaire covering sociodemographic details, health knowledge, information seeking behaviors, and decision-making processes. Association between MHL and pregnancy outcomes were analyzed using descriptive statistics and chi-square tests.

**Results:**

The findings revealed that Maternal health literacy (MHL) was inadequate in majority of participants (65.8%). Preterm birth (40.4%) and low birth weight (34.9%) were the most frequent adverse outcomes, while gestational diabetes was the most common prenatal complication. MHL was significantly associated with husband’s education (*p* = 0.033), maternal employment status (*p* = 0.005), gestational diabetes (*p* = 0.027) and preterm delivery (*p* = 0.036). No significant association was observed between maternal health literacy and postnatal complications (*p* > 0.05).

**Conclusion:**

This study signifies the importance of maternal health education, its impact on outcomes and reveals significant gaps in maternal health literacy. These results highlighted the need to consider maternal health literacy in antenatal care strategies to improve maternal and neonatal health outcomes.

**Supplementary Information:**

The online version contains supplementary material available at https://doi.org/10.1186/s12884-026-09512-4.

## Introduction

Health literacy is defined as an individual’s ability to access, understand, and apply health related information and services to make informed decisions that promote and improve their health [1]. Adequate health literacy is particularly crucial for women of reproductive age groups, as it empowers women to have better access of knowledge, and make informed decision related to antenatal care, childbirth and parenting [2, 3].

Globally, maternal health literacy is a major public health concern, particularly in low- and middle-income countries. Structural and cultural barriers, delayed care-seeking, poor knowledge of pregnancy-related information and inadequate use of maternal health services contribute to preventable adverse maternal and neonatal outcomes [4, 5]. Previous studies have shown that low levels of maternal health literacy have been linked to inadequate antenatal care (ANC) attendance, decreased use of trained birth attendants, poor birth preparation, and a higher risk of maternal and newborn morbidity [6, 7].

Pakistan continues to face major challenges with maternal health challenges, with a maternal mortality ratio (MMR) of approximately 186 deaths per 100,000 live births, which remains higher than the global average [7]. Literature has shown that maternal health behaviors in Pakistan are greatly influenced by sociodemographic factors such as low female literacy, poverty, and cultural norms [8, 9] Women’s literacy rates are still lower than those of men, and there are significant differences between rural and urban areas, directly limiting women’s access to health information and maternal essential services [10, 11]. While formal education influences health literacy, maternal health literacy encompasses broader competencies, including the ability to seek, evaluate, and apply health information during pregnancy. Higher maternal education has been found to have a strong correlation with the use of services, such as skilled birth attendance and prenatal care [12].

Despite the recognized importance of maternal health literacy, evidence from Pakistan is limited and fragmented. Moreover, the absence of pregnancy-specific validated measurements tools in local research has further constraint the understanding of maternal health literacy in Pakistan. This study addresses this gap by using the Maternal Health Literacy in Pregnancy (MHELIP) questionnaire, which allows for a thorough evaluation of maternal health literacy and its relationship to clinically significant pregnancy outcomes.

This project intends to assess the level of maternal health literacy among pregnant women and to examine its association with selected maternal and neonatal outcomes at a tertiary care hospital in Karachi. The findings are intended to inform context-specific interventions and influence clinical practice and policy to improve maternal and newborn outcomes in similar resource-constrained contexts by guiding the design of focused interventions and supporting the incorporation of maternal health literacy evaluation into routine prenatal services.

## Methodology

### Study design and setting

This longitudinal study was conducted in the Obstetrics and Gynecology Department of The Indus Hospital, Korangi Campus, Karachi over a six-month period, to determine the level of maternal health literacy among admitted pregnant females and follow participants until delivery to document pregnancy and neonatal outcomes.

### Study population and sampling

A sample size of 386 participants was calculated using the WHO sample size calculator, assuming a 95% confidence level, 5% absolute precision, and an estimated 48.9% prevalence of maternal health literacy among pregnant women [13].

Participants were selected through non-probability consecutive sampling. Pregnant women of any age groups, including both primigravida and multigravida patients with a gestational age of more than 28 weeks who were willing to participate were considered eligible were included in the study. Patients who were in active labor, had mental disabilities, or those who refused to give their consent were excluded.

### Ethical approval

The study was carried out following approval from the Institutional Review Board (IRB) IHHN-IRB #: IHHN_IRB_2023_01_027 of The Indus Hospital and the College of Physicians and Surgeons of Pakistan (CPSP). Informed verbal consent was obtained from all eligible participants at their first antenatal visit after explaining the purpose and procedure of the study.

### Data collection tool and procedure

Eligible participants were interviewed at enrollment during the antenatal period using a validated questionnaire Maternal Health Literacy Inventory in Pregnancy (MHELIP) scale with high internal consistency (Cronbach’s alpha = 0.94), to assess maternal health literacy [13]. The questionnaire consisted of three sections, the first section gathered sociodemographic and health-related information (questions 1–10), while the second section comprised the Maternal Health Literacy Inventory in Pregnancy (MHELIP) scale, covering four domains: maternal health knowledge (questions 1–21), search for maternal health information (questions 22–27), assessment of maternal health information (questions 28–33), and maternal health decision-making and behavior (questions 34–48). These sections were completed during the antenatal period.

Participants were then followed from enrollment until delivery. After childbirth, information related to pregnancy and neonatal outcomes, including mode of delivery, gestational age at delivery and birth weight were recorded at the time of childbirth using hospital records.

A total of 415 participants were initially enrolled. However, 29 participants were excluded from the final analysis as they delivered at healthcare facilities other than Indus Hospital and were therefore, did not complete the study follow-up. Consequently, data from 386 participants were included in the final analysis.

All participants were informed of their right to voluntarily participate or withdraw from the study at any time without any consequences. Confidentiality was strictly maintained, and all collected data were accessible only to the principal investigator. No incentives were provided for participation, and no penalties were imposed for withdrawal.

**Table:** 

Manual for scoring the maternal health literacy inventory in pregnancy (MHELIP)		
Number of items	Minimum possible raw score	Maximum possible raw score
Maternal Health Knowledge	21 (item 1–21)21	105
Search for maternal health information	6 (item 22–27)6	30
Assessment of Maternal Health Information	6 (item 28–33)6	30
Maternal Health Decision Making and Behavior	15 (item 34–38)	75

To calculate each subscale or total score for the MHELIP, first we added raw scores and linearly transferred it to a score from 0 to 100 using the following formula. 

  

$$\begin{aligned}&Score\\&=\frac{(Raw\;score-Minimum\;possible\;raw\;score)}{Maximum\;possible\;raw\;score-Minimum\;possible\;raw\;score}\\&*100\end{aligned}$$

MHELIP score categorized into 4 categories: inadequate, problematic (which together also defines limited health literacy), sufficient and excellent (desired health literacy):

Inadequate = 0–50.

Problematic = 50.1–66.

Sufficient = 66.1–84.

Excellent = 84.1–100.

As a tertiary care facility, The Indus Hospital serves as a major referral center for high-risk pregnancies and obstetric emergencies across Karachi and its outskirts, which naturally results in a study population with a higher baseline risk for adverse outcomes compared to the general population.

### Data analysis

The data were analyzed using SPSS version 26. Quantitative variables such as age, gestational age and maternal health literacy scores were reported as mean (SD) and median (IQR) based on the normality of the data. The Shapiro-Wilk test was applied to assess the normality of data. Qualitative variables are presented as frequencies and percentages included residence, maternal/paternal education, parity, gravida, occupation, comorbid (DM, HTN, thyroid disorder), pregnancy outcomes (mode of delivery, preterm/term/post term delivery, low birth weight, poor APGAR score, and maternal complication (postpartum hemorrhage, sepsis and vaginal discharge). Maternal health literacy levels were categorized as Inadequate/Problematic/Sufficient/Excellent according to standard MHELIP scoring guidelines. Furthermore, Chi-square / Fischer Exact test was applied for the comparison of MHELIP status with baseline variables considering the p-value < 0.05 as statistically significant.

## Result

**Fig. 1:**
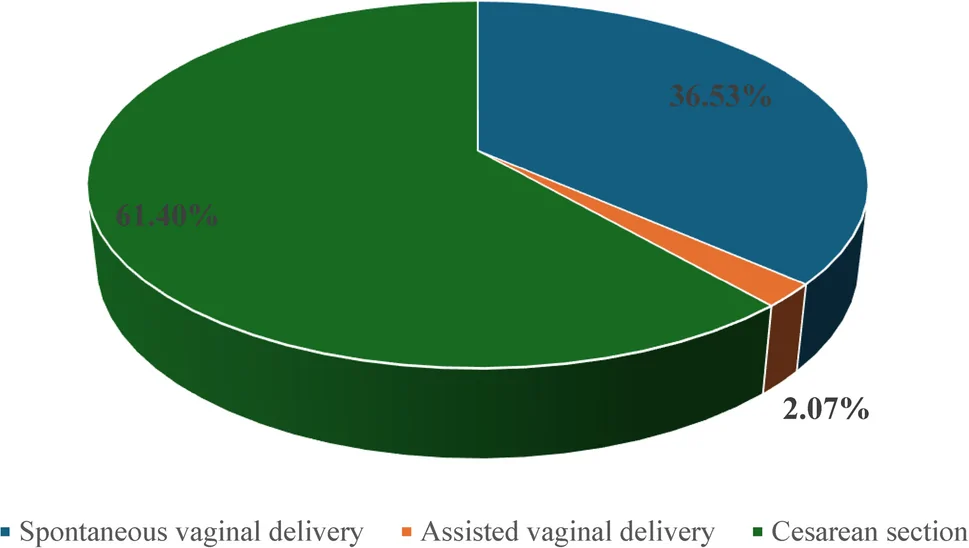
Distribution of mode of Delivery

In this study we enrolled 386 pregnant women. The mean age of women was 27.23 ± 5.13 years. Among 386 women, 249 (64.51%) were multigravida while 177 (45.85%) were multiparous (Table 1). However, mean gestational age at the time of delivery was 37.11 ± 2.25 weeks. Moreover, 237 (61.4%) women delivered through C-section (Fig. 1).

**Table 1:** Distribution of demographic, gravida and parity of study participants

Variables	*n* (%)
Age (Years)	27.23 ± 5.13
Patient Education	
No formal education	30 (7.79)
Primary	31 (8.05)
Secondary	95 (24.68)
Matric	124 (32.21)
Intermediate	83 (21.56)
Graduate	22 (5.71)
Spouse Education	
No formal education	53 (13.73)
Primary	53 (13.73)
Secondary	74 (19.17)
Intermediate	90 (23.32)
Matric	62 (16.06)
Graduate	54 (13.99)
Employment Status	
Employed	73 (18.91)
Unemployed	313 (81.09)
Ethnicity	
Sindhi	67 (17.36)
Punjabi	104 (26.94)
Balochi	67 (17.36)
Pathan	27 (6.99)
Urdu	110 (28.5)
Others	11 (2.85)
Gravida	
Primigravida	137 (35.49)
Multigravida	249 (64.51)
Parity	
Nulliparous	164 (42.49)
Multiparous (1–3 parity)	177 (45.85)
Grand multiparity (> 4 parity)	45 (11.66)

**Fig. 2:**
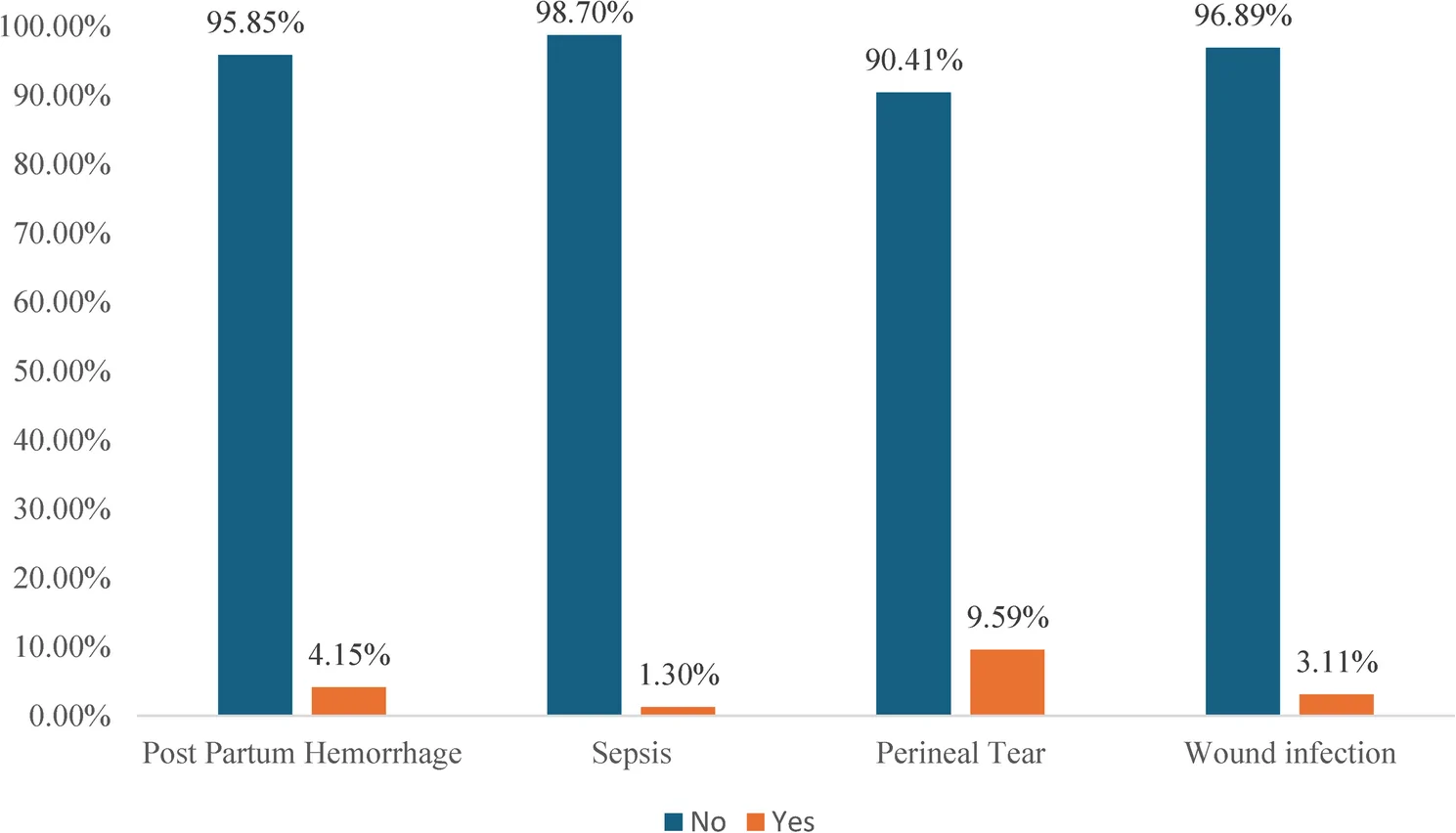
Distribution of post-natal complications

Furthermore, during antenatal period, gestational diabetes was the most common complication observed in 113 (29.27%) women followed by gestational hypertension in 21 (5.44%) and preeclampsia in 11 (2.85%) women (Table 2). In addition, preterm birth was the most common adverse neonatal outcome, observed in 156 (40.40%) followed by low birth weight in 135 (34.97%) and asphyxia in 32 (8.36%) women (Table 3). Subsequently, during post-natal period, perineal tears were observed in 37 (9.6%), post-partum hemorrhage in 16 (4.1%), wound infection in 12 (3.1%) and sepsis in 5 (1.3%) women (Fig. 2).

**Table 2:** Distribution of antenatal complications

Antenatal Complications	*n*(%)
Gestational Diabetes	
No	273 (70.73)
Yes	113 (29.27)
Gestational Hypertension	
No	365 (94.56)
Yes	21 (5.44)
Preeclampsia	
No	375 (97.15)
Yes	11 (2.85)
Eclampsia	
No	384 (99.48)
Yes	2 (0.52)

**Table 3:** Distribution of neonatal outcomes at the time of birth

Neonatal Outcome	*n*(%)
Preterm Birth	
No	230 (59.60)
Yes	156 (40.40)
Low Birth Weight	
No	251 (65.03)
Yes	135 (34.97)
APGAR Score (1 min)	
Normal	349 (91.6)
Mild Asphyxia	23 (6.04)
Severe Asphyxia	9 (2.36)
APGAR Score (5 min)	
Normal	374 (98.16)
Mild Asphyxia	6 (1.57)
Severe Asphyxia	1 (0.26)

**Fig. 3:**
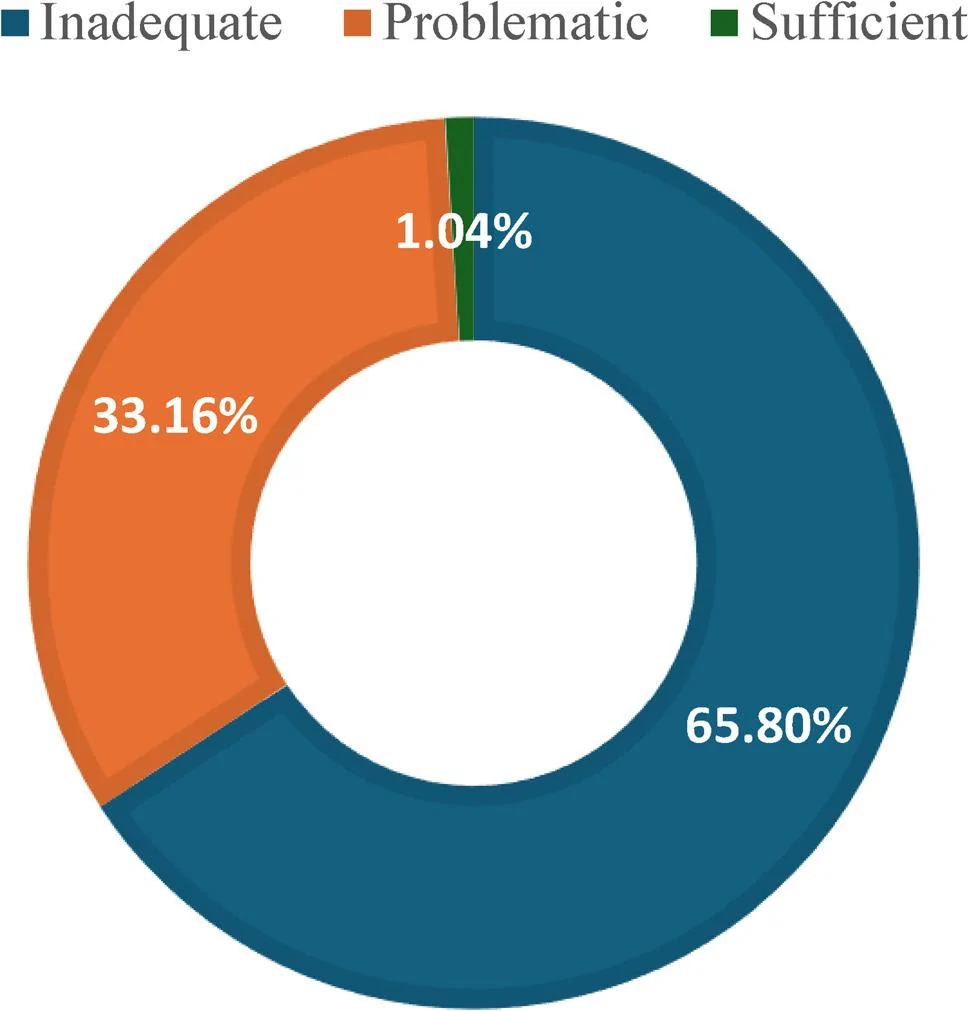
Distribution of maternal health literacy as per maternal health literacy inventory in pregnancy (MHELIP) questionnaire

Figure 3 shows that 254 (65.80%) of the women had inadequate knowledge, followed by problematic in 128 (33.16%) and sufficient knowledge in 4 (1.04%) women. Tables 4 and 5 shows the distribution of maternal, obstetric, and neonatal characteristics according to levels of maternal health literacy. Overall, the majority of mothers fell into the inadequate and problematic health literacy categories, while only a small proportion demonstrated sufficient health literacy. A significant association was found between Maternal health literacy and husband education, employment status, ethnicity, gestational diabetes and preterm delivery. Findings of our study reveals that maternal health literacy was better among women whose husband had matric as compared to no formal education (3.23% vs. 1.89%, p-value 0.033). Similarly, employed women were more likely to have sufficient health literacy as compared to unemployed women (2.74% vs. 0.64%, p-value 0.005). Among antenatal complications, gestational diabetes was significantly associated with maternal health literacy with a high proportion of cases observed among women with inadequate health literacy (75.22% vs. 61.90%, p-value 0.027). In terms of neonatal outcomes, preterm delivery showed a statistically significant association with problematic maternal health literacy levels (39.10% vs. 29.13%, p-value 0.036).

**Table 4:** Association of maternal literacy with demographic, gravida and parity

Independent Variables	Maternal Health Literacy			*p*-value
	Inadequate *n*(%)	Problematic *n*(%)	Sufficient *n*(%)	
Age (Years)				
≤ 25	110 (67.48)	52 (31.9)	1 (0.61)	0.690
> 25	144 (64.57)	76 (34.08)	3 (1.35)	
Mother Education				
No formal education	24 (80)	5 (16.67)	1 (3.33)	0.095
Primary	21 (67.74)	10 (32.26)	0 (0)	
Secondary	58 (61.05)	35 (36.84)	2 (2.11)	
Matric	90 (72.58)	34 (27.42)	0 (0)	
Intermediate	50 (60.24)	32 (38.55)	1 (1.2)	
Graduate	10 (45.45)	12 (54.55)	0 (0)	
Husband Education				
No formal education	41 (77.36)	11 (20.75)	1 (1.89)	0.033
Primary	35 (66.04)	18 (33.96)	0 (0)	
Secondary	48 (64.86)	26 (35.14)	0 (0)	
Intermediate	56 (62.22)	34 (37.78)	0 (0)	
Matric	47 (75.81)	13 (20.97)	2 (3.23)	
Graduate	27 (50)	26 (48.15)	1 (1.85)	
Mother Employment Status				
Employed	37 (50.68)	34 (46.58)	2 (2.74)	0.005
Unemployed	217 (69.33)	94 (30.03)	2 (0.64)	
Ethnicity				
Sindhi	41 (61.19)	24 (35.82)	2 (2.99)	0.012
Punjabi	64 (61.54)	40 (38.46)	0 (0)	
Balochi	35 (52.24)	31 (46.27)	1 (1.49)	
Pathan	17 (62.96)	10 (37.04)	0 (0)	
Urdu	89 (80.91)	20 (18.18)	1 (0.91)	
Others	8 (72.73)	3 (27.27)	0 (0)	
Gravida				
Primigravida	91 (66.42)	44 (32.12)	2 (1.46)	0.799
Multigravida	163 (65.46)	84 (33.73)	2 (0.8)	
Parity				
Nulliparous	105 (64.02)	56 (34.15)	3 (1.83)	0.683
Multiparous	120 (67.8)	56 (31.64)	1 (0.56)	
Grand multiparity	29 (64.44)	16 (35.56)	0 (0)	

**Table 5:** Association of maternal literacy with ANC complications, neonatal outcomes and PNC complications

Independent Variables	Maternal Health Literacy			*p*-value
	Inadequate *n*(%)	Problematic *n*(%)	Sufficient *n*(%)	
Antenatal Complications				
Gestational Diabetes				
No	169 (61.9)	100 (36.63)	4 (1.47)	0.027
Yes	85 (75.22)	28 (24.78)	0 (0)	
Gestational Hypertension				
No	243 (66.58)	118 (32.33)	4 (1.1)	0.327
Yes	11 (52.38)	10 (47.62)	0 (0)	
Preeclampsia				
No	248 (66.13)	123 (32.8)	4 (1.07)	0.653
Yes	6 (54.55)	5 (45.45)	0 (0)	
Eclampsia				
No	252 (65.63)	128 (33.33)	4 (1.04)	0.593
Yes	2 (100)	0 (0)	0 (0)	
Neonatal Outcomes at the time of Birth				
Preterm Delivery				
No	162 (70.43)	67 (29.13)	1 (0.43)	0.036
Yes	92 (58.97)	61 (39.1)	3 (1.92)	
Low Birth Weight				
No	170 (67.73)	79 (31.47)	2 (0.8)	0.492
Yes	84 (62.22)	49 (36.3)	2 (1.48)	
APGAR Score (1 min)				
Normal	228 (65.33)	117 (33.52)	4 (1.15)	0.574
Mild Asphyxia	17 (73.91)	6 (26.09)	0 (0)	
Severe Asphyxia	4 (44.44)	5 (55.56)	0 (0)	
APGAR Score (5 min)				
Normal	247 (66.04)	123 (32.89)	4 (1.07)	0.122
Mild Asphyxia	1 (16.67)	5 (83.33)	0 (0)	
Severe Asphyxia	1 (100)	0 (0)	0 (0)	
Post-natal Complications				
Post-Partum Hemorrhage				
No	243 (65.68)	123 (33.24)	4 (1.08)	0.899
Yes	11 (68.75)	5 (31.25)	0 (0)	
Sepsis				
No	249 (65.35)	128 (33.6)	4 (1.05)	0.268
Yes	5 (100)	0 (0)	0 (0)	
Perineal Tears				
No	225 (64.47)	120 (34.38)	4 (1.15)	0.218
Yes	29 (78.38)	8 (21.62)	0 (0)	
Wound Infection				
No	247 (66.04)	123 (32.89)	4 (1.07)	0.777
Yes	7 (58.33)	5 (41.67)	0 (0)	

Women with inadequate health literacy demonstrated the highest rates of gestational diabetes (75.2% of GDM cases) compared to those without GDM (61.9%), while preterm delivery was most concentrated among women with problematic literacy (39.1%) compared to those who delivered at term (29.1%). Although the small number of participants in the sufficient literacy category (*n* = 4) limits conclusions about a full gradient, the data broadly indicates that limited health literacy is associated with a higher burden of adverse pregnancy outcomes in this cohort.

## Discussion

This study demonstrated a substantial burden of limited health literacy among pregnant women, with vast majority classified as inadequate (65.8%), or problematic (33.16%), with only a small fraction of women had sufficient maternal health literacy (1.04%). These findings are consistent with reports from other low-and middle- income countries, including studies from Laos and Egypt, which similarly identified low levels of sufficient maternal health literacy [14, 15]. This correlates with the global evidence showing that limited health literacy among pregnant women remains a significant concern, affecting health behaviors and outcomes worldwide [16]. The high prevalence of preterm birth (40.4%) and low birth weight (34.9%) observed in this cohort likely reflects selection bias inherent to a tertiary care setting. Many participants were referred to due to existing complications or lack of access to primary prenatal care elsewhere, rather than being representative of the general pregnant population.

According to a scoping review, maternal health literacy was significantly influenced by socioeconomic factors and demographic factors, including income, education and impacts the use of prenatal care, skilled delivery attendance, and postnatal services [17]. Our present study demonstrated that higher maternal health literacy was more frequently observed among women who were employed (*p* = 0.005) and those whose husbands had higher education level (*p* = 0.033). These findings align with existing literature showing that family level of education and women’s economic participation are linked to improve the access to health information, ability to understand, and enhance decision-making capacity [18, 19]. While formal education does not equate to health literacy, it likely facilitates the development of skills required to seek, understand, and apply health information during pregnancy.

The significant association between husband’s education and maternal health literacy (*p* = 0.033) highlights the role of the spouse as a critical mediator in health information seeking. In a cultural context where healthcare decisions are often collaborative or male-led, an educated husband may be more likely to support his wife in accessing health resources and understanding medical instructions. Similarly, the higher levels of sufficient health literacy among employed women (2.74% vs. 0.64%) suggest that professional environments provide exposure to broader social networks and information sources outside the home. Employment also fosters financial autonomy and confidence, empowering women to navigate hospital systems more effectively. Taken together, these findings suggest that maternal health literacy is deeply embedded in household socioeconomic structure, and interventions must consider a family-centered approach involving spouses and addressing barriers faced by unemployed women. Ethnic disparities in maternal health literacy observed in this study (*p* = 0.012) further demonstrate the impact of socio-cultural context factors on health information access and utilization. Previous reviews have similarly reported variation in health literacy associated with socioeconomic and demographic features, suggesting that culturally and linguistically appropriate health communication strategies are essential, particularly in diverse populations [9, 20].

Gestational diabetes (GDM) was observed as the most frequent antenatal complication and was significantly associated with lower levels of maternal health literacy (75.2%, *p* = 0.027), affecting nearly one-third of patients (29.3%). This prevalence reflects the increasing burden of metabolic diseases during pregnancy. Veerasetty NK et. el conducted a study among pregnant women with GDM demonstrated that women with adequate health literacy showed better self-management abilities, dietary adherence, and good glycemic control which are essential to optimal GDM management [21, 22]. Poor glycemic control during pregnancy poses both immediate and long-term risk to both mother and children, including maternal metabolic disorders [23] and neurodevelopmental challenges [24, 25]. The observed association in this study highlights the importance of maternal understanding and engagement in diabetes management during pregnancy to reduce adverse maternal and offspring outcomes.

The mechanistic link between low MHL and gestational diabetes (*p* = 0.027) likely stems from a comprehension-action gap. Women with inadequate MHL may struggle to interpret nutritional labels, understand glycemic monitoring, or adhere to pharmacological regimens, leading to poor glycemic control, a known physiological trigger for maternal and fetal complications.

=The most frequent unfavourable neonatal outcome was preterm birth (40.4%) and was significantly associated with problematic maternal health literacy (*p* = 0.036). This finding is consistent with a systemic review indicating that low maternal health literacy is associated with preterm birth and low birth weight [26]. Although low birth weight was also prevalent in the study group (34.9%), their associations with literacy were not statistically significant, suggesting that other factors may play a stronger role. While postnatal complications were not significantly associated with maternal health literacy in this study, previous research suggests that better maternal literacy improves postnatal selfcare, early recognition of danger signs, and timely health service utilization, thereby reducing morbidity [27].

Regarding preterm delivery (*p* = 0.036), the mechanism may involve delayed danger-sign recognition. Mothers with limited MHL may not identify early symptoms of preterm labor or urinary tract infections, a common preventable cause of prematurity, resulting in delayed care-seeking. Notably, preterm delivery in this study was most concentrated in the problematic literacy category (39.1%) rather than the inadequate category, suggesting the relationship is not strictly linear and may reflect other mediating factors.

Overall, the high prevalence of limited maternal health literacy observed in this study is concerning and reflect broader systemic gaps in prenatal education and patient-centered communication. These findings suggest that maternal heath literacy may represent an efficient and potentially modifiable factors associated with keys pregnancy outcomes in resource limited settings.

Potential confounding factors must also be acknowledged. Socioeconomic status, particularly household income and access to transport, may act as a confounder, low SES is independently associated with both limited MHL and with poor nutritional intake or physical stress, both of which increase the risk of gestational diabetes and preterm birth. While this study accounted for employment and education, unmeasured social determinants of health may partially explain the observed associations.

### Strength and limitations

This study has several strengths, including an adequate sample size, use of a validated pregnancy-specific health literacy tool, and prospective follow-up until delivery. However, several limitations should be acknowledged. The study was conducted at a single tertiary care hospital, which may limit its generalizability to other settings. Additionally, reliance on self-reported measures of health literacy may introduce reporting bias. Subsequently, MHELIP questionnaire has not been validated in the Pakistani context. Although the scale demonstrated excellent internal consistency in our study, but future validation studies are recommended to validate the MHELIP scale in the Pakistani context.

These results highlight the urgent need to integrate structured maternal health literacy into routine prenatal care. There are many techniques and strategies that could contribute to increase comprehension and engagement such as, use of simplified educational materials, encourage spouse participation, tailored cultural issues, and introduction of counselling adapted to literacy levels. Strengthening maternal health literacy can improve both maternal and newborn health outcomes by lowering avoidable problems including gestational diabetes and preterm birth in resource-limited settings. Strengthening general female literacy is essential to increasing health literacy from a broader public health viewpoint because women’s capacity to acquire, comprehend, and act upon health information throughout their lives is supported by core reading, numeracy, and communication abilities. While the high rate of adverse outcomes provides a robust environment for studying MHL associations, it may limit the generalizability of absolute prevalence rates to lower-risk community or primary care settings.

## Conclusion

This study highlights the high burden of inadequate and problematic maternal health literacy among pregnant women in a tertiary care setting in Pakistan. Lower maternal health literacy was significantly associated with sociodemographic factors, including husband’s education, maternal employment, and ethnicity, as well as with adverse pregnancy outcomes such as gestational diabetes and preterm delivery. Integrating maternal health literacy assessment and tailored communication strategies into routine antenatal care can help break cycles of poor maternal and child health in similar resource constraint contexts

## Supplementary Information

Supplementary Material 1

## Data Availability

The datasets used and/or analyzed during the current study are available from the corresponding author on reasonable request.
