# Supplementary material for: Evaluation of maternal health literacy and its impact on pregnancy outcomes: A longitudinal study in a tertiary care hospital, Karachi
Source: BMC Pregnancy Childbirth. 2026 Jul 1;26:1033. doi: 10.1186/s12884-026-09512-4 (PMC13587492; doi:10.1186/s12884-026-09512-4)
Supplement: Supplementary file 1 [file 12884_2026_9512_MOESM1_ESM.docx]

**Questionnaire:**

| **Mr. no:** | **_______________** |
| --- | --- |
| **Serial number:** | **_______________** |
| **Age** | **_______________** |
| **Patients’ Education status:** | 1. **No formal education**  - **able to read** - **not able to read**  2. **Primary** 3. **Secondary** 4. **Intermediate** 5. **Graduate.** |
| **Comorbid conditions:** | 1. **Diabetes** 2. **Hypertension** 3. **Thyroid disorder** 4. **Others_____________** |
| **Gravida:** | **Primigravida**  **Multigravida** |
| **Parity** | **Nulliparous**  **Multiparous** |
| **Current Gestational age** | **____________________** |
| **Spouse Education:** | 1. **No formal education**  - **able to read** - **not able to read**  2. **Primary** 3. **Secondary** 4. **Intermediate** 5. **Graduate** |
| **Occupation:** | **Employed**  **Unemployed** |
| **Ethnicity** | 1. **Sindhi** 2. **Punjabi** 3. **Balochi** 4. **Pathan** 5. **Urdu speaking** 6. **Others** |
|  |  |

**The maternal health literacy inventory in pregnancy (MHELIP)**

| **Item** | **I don't know at all.**  **(1)** | **I know a little**  **(2)** | **I know something.**  **(3)** | **I know a lot.**  **(4)** | **I know fully.**  **(5)** |
| --- | --- | --- | --- | --- | --- |
| **1.I know natural physical changes during pregnancy.** |  |  |  |  |  |
| **2.I know natural psychological changes during pregnancy.** |  |  |  |  |  |
| **3.I know proper nutrition during pregnancy.** |  |  |  |  |  |
| **4.I know personal health care.** |  |  |  |  |  |
| **5.I know Proper activity and status in pregnancy.** |  |  |  |  |  |
| **6.I know proper exercise during pregnancy.** |  |  |  |  |  |
| **7.I know pregnancy supplements (vitamins).** |  |  |  |  |  |
| **8.I know the appropriate referral timing for pregnancy examinations (visits).** |  |  |  |  |  |
| **9.I know diagnostic examination (ultrasound and tests) of maternal and fetal health in pregnancy.** |  |  |  |  |  |
| **10.I know the acceptable and normal amount of weight gain during pregnancy.** |  |  |  |  |  |
| **11.I know common pregnancy problems such as nausea, vomiting, lower back pain.** |  |  |  |  |  |
| **12.I know injecting safe (allowed) vaccines during pregnancy.** |  |  |  |  |  |

| **13.I know the proper sexual relation during pregnancy.** |  |  |  |  |  |
| --- | --- | --- | --- | --- | --- |
| **14.I know the normal number of fetal movements.** |  |  |  |  |  |
| **15.I know the factors affecting fetal health such as photography, medications, chemicals such as Botox, etc.** |  |  |  |  |  |
| **16.I know risk signs in pregnancy.** |  |  |  |  |  |
| **17.I know pregnancy disease symptoms such as gestational diabetes, high blood pressure in pregnancy and other diseases.** |  |  |  |  |  |
| **18.I know childbirth such as the advantages and disadvantages of each of the natural delivery methods and cesarean section and their associated care.** |  |  |  |  |  |
| **19.I know the methods of pain relief in virginal delivery.** |  |  |  |  |  |
| **20.I know neonatal and infant care in the postpartum period.** |  |  |  |  |  |
| **21.I know the required postpartum care of mother.** |  |  |  |  |  |
| **Item** | **Not**  **at all**  **(1)** | **Rarely**  **(2)** | **Sometimes**  **(3)** | **most often the times**  **(4)** | **Always**  **(5)** |
| **22.I acquire information from written materials such as books, educational notes, pamphlets, and medication brochures.** |  |  |  |  |  |
| **23.I acquire information from radio and television.** |  |  |  |  |  |

| **24.I acquire information from internet sources such as websites, Instagram, and telegram.** |  |  |  |  |  |
| --- | --- | --- | --- | --- | --- |
| **25.I acquire information from other pregnant women.** |  |  |  |  |  |
| **26.I acquire information from family, friends, and acquaintances.** |  |  |  |  |  |
| **27.I acquire information from healthcare professionals such as a physician or midwife.** |  |  |  |  |  |
| **28.It is easy for me to read and pronounce pregnancy-related vocabulary from information sources such as books, educational booklets, internet, telegram, and Instagram.** |  |  |  |  |  |
| **29.The information obtained from different sources of information is understandable for me.** |  |  |  |  |  |
| **30.I know valid and verified sources for getting the right pregnancy related information.** |  |  |  |  |  |
| **31. I ask of the doctor or midwife to make sure pregnancy related information.** |  |  |  |  |  |
| **32.I Evaluate the accuracy of pregnancy related information obtained from online sources such as websites, Instagram, and telegram.** |  |  |  |  |  |
| **33.I Evaluate the accuracy of pregnancy related information obtained from friends and relatives** |  |  |  |  |  |
| **34. I can control/manage physical and psychological changes in pregnancy.** |  |  |  |  |  |
| **35.I implement a proper diet for pregnancy.** |  |  |  |  |  |

| **36.I implement necessary measures for personal health care during pregnancy.** |  |  |  |  |  |
| --- | --- | --- | --- | --- | --- |
| **37.I adhere to the principles of activity and proper condition during pregnancy.** |  |  |  |  |  |
| **38.I take pregnancy supplements as prescribed by doctor or midwife.** |  |  |  |  |  |
| **39.I consult with the doctor or midwife for taking any type of medication during pregnancy (chemical and herbal).** |  |  |  |  |  |
| **40.I attend prenatal care (examinations) as scheduled.** |  |  |  |  |  |
| **41.I Perform ultrasound and tests in pregnancy recommended by healthcare professionals such as doctor or midwife.** |  |  |  |  |  |
| **42.I monitor and control weight gain during pregnancy.** |  |  |  |  |  |
| **43.I use the appropriate methods of sexual relations during pregnancy.** |  |  |  |  |  |
| **44.I avoid taking actions that are harmful to pregnancy.** |  |  |  |  |  |
| **45.I see the doctor or midwife as soon as possible when any signs of danger in pregnancy are observed.** |  |  |  |  |  |
| **46.I ask the doctor or midwife for further explanation if the information and recommendations are not clear enough.** |  |  |  |  |  |
| **47.I participate in decision making about pregnancy issues with the doctor or midwife (providing personal opinions).** |  |  |  |  |  |
| **48.I pay attention to the accuracy and appropriateness of information given to other pregnant women.** |  |  |  |  |  |
| **Maternal Health Knowledge: 1-21 question**  **Search for maternal health information: 22-27 question**  **Assessment of Maternal Health Information:28-33 question**  **Maternal Health Decision Making and Behavior:34-48 question** | | | | | |

**Pregnancy outcome:**

| **Delivery type:** | - **Spontaneous vaginal delivery** - **Assisted vaginal delivery** - **Intra-partum caesarean delivery** - **Planned Cesarean section** |
| --- | --- |
| **Gestational age at birth** | - **____________________** |
| **Outcome** | - **Preterm delivery** - **Term delivery** - **Post term delivery** |
| **Birth weight** | - **____________________** |
| **Small for gestational age** | - **____________________** |
| **Large for gestational age** | - **____________________** |
| **5 min Apgar score** | - **____________________** |
| **Maternal complications** | - **Hemorrhage** - **Sepsis** - **vaginal discharge** |
